# Efficacy of Chemicals for the Potential Management of the Queensland Fruit Fly *Bactrocera tryoni* (Froggatt) (Diptera: Tephritidae)

**DOI:** 10.3390/insects8020049

**Published:** 2017-05-09

**Authors:** Olivia L. Reynolds, Terrence J. Osborne, Idris Barchia

**Affiliations:** 1 Graham Centre for Agricultural Innovation (New South Wales Department of Primary Industries and Charles Sturt University), Elizabeth Macarthur Agricultural Institute, Private Bag 4008, Narellan, NSW 2567, Australia; 2 New South Wales Department of Primary Industries, Biosecurity and Food Safety, Elizabeth Macarthur Agricultural Institute, Private Bag 4008, Narellan, NSW 2567, Australia; terry.osborne@dpi.nsw.gov.au; 3 New South Wales Department of Primary Industries, Chief Scientist’s Branch, Elizabeth Macarthur Agricultural Institute, Private Bag 4008, Narellan, NSW 2567, Australia; idris.barchia@dpi.nsw.gov.au

**Keywords:** pesticide, insecticide, dimethoate, fenthion, alpha-cypermethrin, clothianidin, summerfruit, area wide management, integrated pest management

## Abstract

This study investigated alternative in-field chemical controls against *Bactrocera tryoni* (Froggatt). Bioassay 1 tested the mortality of adults exposed to fruit and filter paper dipped in insecticide, and the topical application of insecticide to adults/fruit. Bioassay 2 measured the mortality of adults permitted to oviposit on fruit dipped in insecticide and aged 0, 1, 3, or 5 days, plus the production of offspring. Bioassay 3 tested infested fruit sprayed with insecticide. The field bioassay trialed the mortality of adults exposed to one- and five-day insecticide residues on peaches, and subsequent offspring. Abamectin, alpha-cypermethrin, clothianidin, dimethoate (half-label rate), emamectin benzoate, fenthion (half- and full-label rate), and trichlorfon were the most efficacious in bioassay 1, across 18 tested insecticide treatments. Overall, the LT50 value was lowest for fenthion (full-label rate), clothianidin, and alpha-cypermethrin. Fenthion, emamectin benzoate, and abamectin had the greatest effect on adult mortality and offspring production. Infested fruit treated with acetamiprid, fenthion, and thiacloprid produced no/very few offspring. Alpha-cypermethrin demonstrated good field efficacy against adults (one day post treatment: 97.2% mortality, five day post treatment: 98.8% mortality) and subsequent offspring (100% across one and five day post treatments), comparable to that of fenthion (full-label rate) (100% mortality for offspring and adults across both post treatments). Alpha-cypermethrin is a possible alternative to fenthion against *B. tryoni*; as a pyrethroid, it may not be desirable if adjunct biological control is imperative. Thiacloprid and Acetamiprid may be useful as a post-harvest treatment.

## 1. Introduction

The genus *Bactrocera* (Diptera: Tephritidae) includes approximately 500 described species, most of which are endemic to Southeast Asia and South Pacific islands [1]. Most *Bactrocera* spp. live in subtropical or tropical rain forests and pose no risk to agriculture. However, approximately 70 polyphagous *Bactrocera* species attack commercial fruit and vegetable crops, and are a serious obstacle to international trade [2,3]. In Australia, the Queensland fruit fly, *Bactrocera tryoni* (Froggatt), is the most significant biosecurity pest affecting Australia’s $9 billion-plus per annum horticultural industry, including both domestic and international markets. This species attacks nearly all fruit and many vegetable crops, with over 240 reported hosts [4]. In areas where *B. tryoni* is endemic, intensive in-field control must ensue to ensure high quality produce, with additional postharvest treatments sometimes required to meet the phytosanitary requirements of importing markets. The majority of domestic and international markets have nil tolerance for *B. tryoni*.

Until recently, the organophosphates dimethoate and fenthion have formed a part of many control programs where *B. tryoni* is endemic [5,6,7], or to meet the market access requirements of Australian states where *B. tryoni* is absent (e.g., Tasmania, South Australia, Western Australia) under the Interstate Certification Assurance (ICA) scheme [8]. Both insecticides are partially systemic in the plant and display a moderate mammalian toxicity [9], although fenthion is extremely toxic to birds.

Dimethoate and fenthion were first recommended as cover sprays in the early-mid 1960s [10,11]. O’Loughlin [12] demonstrated that cover sprays with dimethoate and fenthion killed fruit fly larvae developing in the fruit, as well as adult flies. Although concerns with fenthion resulted in a reduction in its use during the 1970s [11], the lack of alternatives led to its reincorporation and continued use [13]. In 2004, the Australian Pesticides and Veterinary Medicines Authority (APVMA) commenced a review of dimethoate use in Australia [14], effectively prohibiting the pre-harvest use of dimethoate on over 60 horticultural crops [15], with the suspension of certain horticultural products that continued until at least 5 April 2017 [16]. Likewise, following the finalisation of the review of fenthion on 16 October 2014 [17], prohibitive and restricted crop uses were covered under permit numbers, PER 13841 (all states and territories except Western Australia (WA)) [18], PER 13840 (WA) [19], and PER 14654 [20], which were extended until October 2015, representing the end of the phase out period for this product. Similarly, the permit for the use of fenthion home garden and horticultural products for *B. tryoni* expired in mid October 2015 [21]. The review of fenthion was completed on 2 November 2015 [17]. All active approvals and product registrations have been cancelled; products containing fenthion may no longer be used or supplied in Australia. Alternate pre-harvest options to replace dimethoate and fenthion for fruit fly control are listed on the APVMA website [22]; however, many are largely ineffective.

There is therefore an urgent need to identify alternative field control options; one of the most immediate is to test alternative chemical controls. An ideal insecticide for the control of *B. tryoni* should be systemic, residual, and highly effective against immatures and adults, while minimising impacts on the environment and human health.

This project has established alternative chemical controls for *B. tryoni* that may offer a viable replacement for dimethoate and fenthion in the field. Specifically, this study identified effective chemical control options for *B. tryoni*, which may form part of an integrated pest management program, ensuring continued market access for domestic and international trade of host fruit commodities.

## 2. Materials and Methods

### 2.1. Insects

*Bactrocera tryoni* were obtained as adults from the fruit fly rearing facility, Central Coast Primary Industries Centre, University of Newcastle Ourimbah Campus, North Loop Road, Ourimbah, New South Wales (NSW), Australia, where they were reared under standard conditions of 26 ± 1 °C, 65% ± 15% relative humidity, and a photoperiod of 14:10 [L:D] h. The flies used in the trials had been held in colony for < one year. Upon emergence, flies were provided with yeast hydrolysate (YH) (MP Biomedical, Auburn, OH; 60% protein) as a source of protein, sugar as a source of carbohydrate, and water *ad libitum*, and permitted to mate. Flies aged 7–10 days were used in the bioassays as detailed below. All laboratory bioassays were conducted at the Elizabeth Macarthur Agricultural Institute, Menangle, NSW, Australia, under standard conditions, except for bioassay 1, which was conducted in a well-ventilated roofed outdoor laboratory. Throughout the duration of bioassay 1, the minimum (1800–0600) temperatures ranged from 14.8–27.9 °C and the maximum (0600–1800) temperatures ranged from 15.0–36.1 °C, while the minimum (1800–0600) relative humidity ranged from 44%–100%, the maximum (0600–1800) humidity ranged from 33%–99%, and the photoperiod was approximately 14:10 [L:D]. Throughout the duration of the field assay, minimum (1800–0600) temperatures ranged from 10.2–27.4 °C and the maximum (0600–1800) temperatures ranged from 10.2–33.5 °C, while the minimum (1800–0600) relative humidity ranged from 30%–100%, the maximum (0600–1800) humidity ranged from 21%–100%, and the photoperiod was approximately 14:10 [L:D].

### 2.2. Laboratory Bioassays

Bioassays were conducted using insecticide products either registered for fruit fly control in Australia and/or for use on fruits against other insect pests in at least one Australian state. The only exception was the DC-092 Coded molecule supplied by Bayer Crop Science, which was a new, previously untested formulation against *B. tryoni*.

#### 2.2.1. Laboratory Bioassay 1: Mortality of Adults

Twenty flies (1:1 sex ratio) were placed inside a 500 mL paper container covered with a fly proof mesh and provided with food (YH and sugar *ad libitum*) and water for 1–2 h prior to trial commencement.

Eighteen treatments (fenthion was used at both a half-label rate and full-label rate) (Table 1) were tested for their effect on adult *B. tryoni* mortality at the label rate, or at the rate recommended by the supplier (except for the half-label rate dimethoate and fenthion) using three application techniques: (i) a whole fruit (nectarine) dipped into the insecticide/water for 2–3 s and allowed to air dry before being placed in the container with the flies, (ii) insecticide/water misted onto adult flies until droplets appeared (topical application), and (iii) filter paper dipped in insecticide and permitted to air dry before being placed on the base of the container.

Flies were exposed to a single treatment per container. Fly mortality was checked and recorded at 5, 15, 30, 60, 120, 240, 480, and 1440 min after exposure. The trial was temporally replicated three times.

Commercially grown nectarines were purchased from a local fruit shop (Camden Fruit Barn, Elderslie, Australia) and used in the first replication. An organic fruit supplier (nectarines; Duralgai Horticultural P/L, Lake Boga, Victoria, Australia) was sourced for replication two and three. Each whole fruit was individually rinsed under cold running water for 20–30 s and allowed to dry completely before use.

#### 2.2.2. Laboratory Bioassay 2: Oviposition and Mortality of Adults

Seven treatments including abamectin, acetamiprid, clothianidin, emamectin benzoate, trichlorfon, fenthion (half-rate), a standard (fenthion; full-rate), and a control (water) were chosen, based on the results of bioassay 1, together with the inclusion of the two best performing neonicotinoids (clothianidin and acetamiprid), which, when compared with the organophosphates and carbamates, cause less toxicity to birds and mammals than to insects. Pesticides were used at the same rate as detailed in Table 1.

Twenty flies (1:1 sex ratio) were placed inside a 30 × 30 × 30 cm mesh cage (Bugdorm, Taiwan) with food (YH and sugar *ad libitum*) and water for 1–2 h prior to trial commencement. Each treatment comprised three whole rinsed organic nectarines (as in bioassay 1) that were dipped in water (control) or one of the seven chemical treatments above for 2–3 s and allowed to air dry. Once the whole fruit was dry, it was placed on a shallow Petri dish (5 cm in diameter) in the centre of the cage, with a piece of filter paper underneath. The residual effect of the insecticide on the fruit was tested by dipping the fruit and allowing it to age for 0, 1, 3, and 5 days before the fly challenge. This was timed so that all treatments were trialled at the same time, i.e., the residual fruit was dipped in each treatment 5, 3, and 1 day prior to trial commencement. The trial was temporally replicated three times.

Fly mortality was checked and recorded at 5, 15, 30, 60, 120, 240, 480, and 1440 min after initial exposure, and then every 24 h for 72 h. After 72 h, the fruit was placed individually above moistened vermiculite (four vermiculite: one water) on a container covered with fine mesh, allowing the passage of juice into the container, but excluding the larvae entering the container. After approximately 7–10 days, the vermiculite was gently sieved and the number of pupae was counted. The fruit were then dissected to determine if there were any remaining larvae. If larvae were found, the fruit was held until all larvae had pupated or were dead. Counted pupae were held in moistened vermiculite until enclosion. All emerging adults were counted, and any deformities were noted.

#### 2.2.3. Laboratory Bioassay 3: Mortality of Offspring in *B. tryoni* Infested Fruit

Six insecticides, a control, and a standard were tested for their effect on egg/larval mortality. Twelve mated flies (1:1 sex ratio) were placed inside each of twenty-four, 30 × 30 × 30 cm mesh cages with food and water, as described for bioassay 2. Three whole rinsed organic nectarines (as per bioassay 1) were placed in each cage. After 48 h, the fruit was removed and sprayed with either: (i) water (control); (ii) fenthion; full-rate (standard); (iii) fenthion; half-rate; iv) emamectin benzoate; (v) clothianidin; (vi) acetamiprid; (vii) imidicloprid; or (viii) thiacloprid for 2–3 s and allowed to air dry on a piece of filter paper. The fruit was then placed individually above moistened vermiculite and the number of offspring (dead larvae, pupae, and adults) was counted, as per bioassay 2. The trial was temporally replicated three times.

Some of the pesticide labels specified the use of a wetting agent for stone fruit (Table 1). Wetting agents were not used in the laboratory bioassays for any of the chemicals tested, although a surfactant was used in the field trial for clothianidin (see below).

### 2.3. Field Bioassay

#### 2.3.1. Pesticide and Trial Fruit

A temporally replicated field trial at Wanaka Orchard, Oakdale, NSW 2570, containing citrus and stone fruit, was conducted to determine the efficacy and residual effects of six pesticide treatments, including: (i) fenthion (full-label rate; standard), (ii) fenthion (half-label rate), (iii) emamectin benzoate, (iv) clothianidin (with MAXX Organosilicone surfactant at 50 mL/100 L water), (v) clothianidin (without surfactant), and (vi) alpha-cypermethrin and water (control), at the rate detailed in Table 1.

To exclude wild fly infestation before the fruit started to mature, 20 cm × 40 cm mesh sleeves (Bugdorm, Taiwan) were placed over branches of peach trees, each sleeve enclosing two to four whole fruit. Replicate 1 included 40 mesh sleeves across 10 peach trees (variety “Snow King”), while replicate 2 and 3 included 28 mesh sleeves across 10 peach trees (variety “Spring Snow”). The trees were not treated with any pesticide at least five days prior to the sleeves being placed over the fruit or for the duration of the trial. A 20 m buffer zone of trees surrounded each treatment to minimize drift. Replicate 1 only compared five of the seven treatments, excluding clothianidin (without surfactant) and alpha-cypermethrin, while replicates 2 and 3 included all seven treatments.

Treatments, and the one- or five-day residual period were randomly allocated to each tree/mesh sleeve. When the fruit was mature and susceptible to *B. tryoni*, it was sprayed (as per label rate/instructions) five days prior to the introduction of 20 flies (1:1 sex ratio) into each mesh sleeve. The same occurred for the remaining mesh sleeves; however, the fruit was sprayed with each treatment one day prior to the introduction of flies. This was timed such that the flies for both the 1- and 5-day residual treatments were introduced into the mesh sleeves on the same day. After the flies had been exposed to each treatment for 48 h, the number of dead flies were counted and sexed per mesh sleeve. The fruit was then harvested, labelled, and returned to the laboratory. The temperature, relative humidity, and rainfall in the field were recorded. Rainfall was classified as: (i) “Light”: trace to 0.25 cm/hr, (ii) “Moderate”: 0.26 to 0.76 cm/h, or (iii) “Heavy”: >0.76 cm/h, based on the American Meteorological Society website’s definition of “Rain” [23]. The label for Astound^®^ (alpha-cypermethrin) states that it should not be applied if rain is expected within 6 h of application and for Sumitomo Samurai Systemic Insecticide™ (clothianidin) if “heavy” rain is expected within 48 h. Although there is no information on the labels for either Lebaycid^®^ (fenthion) or Proclaim^®^ (emamectin benzoate) regarding rain, the suppliers indicated that if rain was not expected within 4 h and 2 h, respectively, of chemical treatment, then the product should work to its potential. All fruit were protected from rain for at least 6 h post pesticide treatment.

#### 2.3.2. Fruit Holding

The number of fruit was recorded per mesh sleeve/tree. Individual whole fruit were then placed over moistened vermiculite for pupal recovery and count. The eclosed adults were sexed and counted, and the dead larvae were recorded.

### 2.4. Statistical Analysis

#### 2.4.1. Bioassay 1: Mortality of Adults

As the observations were made at the end of each time period (5, 15, 30, 60, 120, 240, 480, 1440 min), the individual mortality within each period was recorded at the observed time. Prior to analysis, the observed time of an individual insect death was randomly allocated to the time between the last observation time and the recorded time. This individual mortality time is also called the accelerated failure time (AFT). The AFT was analysed using a survival analysis [24]. After an examination of the observed versus fitted survival function on the iterated log (Time), the Weibull distribution was the best fit to the AFT. The survival function considered to fit these data (***t***) is as follows:

*S(**t**)* = exp(−*λ·**t**^α^*)

where *λ* = exp(∑*b*_i_·X_i_); X_i_ is the i^th^ chemical, *b*_i_ is the shape parameter of the i^th^ chemical, and *α* is the scale parameter of the Weibull distribution. The parameter λ measures the instantaneous mortality rate (i.e., the mortality rate per minute).

All of the parameters were estimated using a maximum likelihood estimation technique and the analysis was run via GenStat for Windows using the RSURVIVAL procedure [25]. The length of the exposure time required to kill 50% of the insects (lethal time at 50% mortality or LT50) and the 95% fiducial limits were estimated by an iterative process of fitting the survival function. All significant differences are based on the shape parameter (*b*_i_) estimates and their standard errors; however, only λ, the LT50 estimates, and the 95% fiducial limits are shown in the tables.

The mortality data at 1440 min were analysed using the generalized linear model approach [26], where errors were assumed to follow a binomial distribution and a logit link was used to relate the data to the chemical effects. Prior to analysis, a zero proportion was added with 1/4***n*** and a 100% proportion was deducted by 1/4***n***, where ***n*** is the initial number of insects [27]. If mortality resulted from the water treatment, this data was used to adjust the morality rates from the chemical treatments to account for natural mortality. All parameters were estimated using a residual maximum likelihood (REML) and the treatment effects were compared using the 95% least significant difference intervals.

#### 2.4.2. Bioassay 2: Oviposition and Mortality of Adults

##### Mortality

Observations were made at the end of each time period (5, 15, 30, 60, 120, 240, 480, 1440, 2880, 4320 min). However, to determine the mean percentage mortality at 24 h, the time to 50% mortality, and the mortality rate per minute (λ), the mortality data at 1440, 2880, and 4320 min were analysed using a generalized linear model. The analysis followed a similar process as that employed for the bioassay 1 mortality data at 1440 min.

##### Offspring

Data were fitted with a GLM, where the errors were assumed to follow a Poisson distribution. A square root link function was used and all parameters were estimated using an REML estimation. The parameter estimates of the linear predictor were compared at a 5% probability level of student *t*-distribution.

#### 2.4.3. Bioassay 3: Mortality of Offspring in *B. tryoni* Infested Fruit

Data were fitted with GLM, where the errors were assumed to follow a Poisson distribution. A logarithmic link function was used and all parameters were estimated using a REML estimation. The parameter estimates of the linear predictor were compared at a 5% probability level of student *t*-distribution.

#### 2.4.4. Field Bioassay

A preliminary analysis using GLM showed there was no significant interaction between the sex and treatment effects, and hence, pooled male and female data were analysed. Data (proportion of death) were fitted with a generalized linear mixed model (GLMM), and the error was assumed to be binomially distributed. A logit link function was used to relate the mortality rate to the parameters (Chemical by Spray day) as follows:

Logit(p) = Fixed(Chemical + Spray day + Chemical × Spray day) + Random(Reps + [Reps × Chemical × Spray day] + Sleeves)

In all studies, the parameters were estimated using REML, and the parameter standard error was adjusted by the variance overdispersion. The parameter estimates were then compared using least significant difference (LSD) at the 5% level.

## 3. Results

### 3.1. Bioassay 1

Overall, based on topical and dipped fruit applications (addition of the percentages in Table 2 and Table 3), abamectin, clothianidin, dimethoate (half-label rate), emamectin benzoate, fenthion (half-label rate and full-label rate standard), and trichlorfon were the six most efficacious insecticides (Table 2 and Table 3). However, when filter paper was included (Table 4), alpha-cypermethrin replaced abamectin as the better performing insecticide. As abamectin is derived from a *Streptomyces* spp. and is not long lived in comparison with the pyrethroid alpha-cypermethrin, it is considered the more environmentally-friendly specimen and was chosen to study in bioassay 2.

#### 3.1.1. Fruit Dipping

Dimethoate had the lowest LT50 at 17 min, and acetamiprid, emamectin benzoate, fenthion, and trichlorfon all had LT50s of under 2 h (χ^2^ = 703, df = 16, *p* ≤ 0.001, Table 2). Several compounds, including imidacloprid, did not kill 50% of the flies in 24 h (Table 2).

#### 3.1.2. Topical Insecticide

Alpha-cypermethrin had the lowest LT50 at 5 min, and abamectin, acetamiprid, bifenthrin, fenthion (half- and full-rate), clothianidin, dimethoate, malathion, methomyl, tau-fluvalinate, and trichlorfon all had LT50s of under 1 h, with only cyantraniliprole and the DC-092-coded molecule not reaching LT50 by the completion of the bioassay (χ^2^ = 1973, df = 17, *p* ≤ 0.001, Table 3).

#### 3.1.3. Filter Paper Dipped in Chemical

Trichlorfon had the lowest LT50 at 15 min, and fenthion (half-rate), alpha-cypermehrin, and dimethoate all had LT50s of under 1 h, while clothianidin and fenthion (full-rate) had LT50s of under 2 h (χ^2^ = 1674, df = 17, *p* ≤ 0.001; Table 4). Several compounds, including imidacloprid and thiacloprid, did not kill 50% of the flies in 24 h (Table 4).

### 3.2. Bioassay 2

#### 3.2.1. Adult Mortality

Fenthion (standard and half-label rate), together with emamectin benzoate, had the greatest effect on mortality, which decreased when the residual increased (χ^2^ = 2047, df = 28, *p* ≤ 0.001; Table 5). Conversely, acetamiprid showed the least efficacy against *B. tryoni*, and did not reach half-kills by the completion of the trial for any residual tested.

#### 3.2.2. Offspring

Based on the number of pupae and adults produced, fenthion was the most effective at preventing offspring development, followed by trichlorfon, emamectin benzoate, and abamectin (*F* = 26.16, df = 7, *p* < 0.05; Table 5). Acetamiprid was the least effective, followed by clothianidin.

### 3.3. Bioassay 3

#### Offspring

Fruit infested with *B. tryoni* and then sprayed with insecticide differed amongst the treatments (*F* = 61.46, df = 6; error df = 182, *p* ≤ 0.001, Table 6). Fruit treated with acetamiprid, fenthion (either half or full-label rate), and thiacloprid produced no, or very few, pupae and adults. Conversely, emamectin benzoate produced the highest number of adults, followed by clothianidin and imidicloprid.

### 3.4. Field Bioassay

#### 3.4.1. Adult Mortality

Preliminary analyses revealed there was no significant effect of sex on the mortality of *B. tryoni* within treatments (*F* = 0.23, df = 4, *p* = 0.922) and hence mortality was pooled across both males and females for analyses. The mortality of adult *B. tryoni* exposed to insecticide in the field bioassay differed across treatments (*F* = 71.8, df = 4, error df = 71, *p* ≤ 0.001, Table 7).

#### 3.4.2. Offspring

Pesticide treated fruit produced fewer offspring compared with water treated fruit (Pupae: χ^2^ = 44.87, df = 1, *p* < 0.001; Adult (F1): χ^2^ = 483.6, df = 1, *p* < 0.001; Table 7), except for dead larvae (χ^2^ = 2.52, df = 1, *p* = 0.112). There was a significant effect of the pesticide used at both 1 and 5 day residuals (χ^2^ = 18.19, df = 5, *p* = 0.017; Table 7). The number of surviving adult flies differed among chemical treatments (χ^2^ = 38.43, df = 5, *p* < 0.001; Table 7).

#### 3.4.3. Rainfall

One replicate was removed from the analyses as ‘heavy’ rain (8.4 mm in 1 h) occurred in the 48 h post spray.

## 4. Discussion

The current study demonstrates that alpha-cypermethrin shows a very good field efficacy against both *B. tryoni* adults and their offspring, approaching that of the organophosphate, fenthion. In general, fenthion (full-label rate) demonstrated the greatest efficacy across all trials. However, following the completion of the review of fenthion by the APVMA, for use in *B. tryoni* management, its use and supply is no longer permitted [17]. Although in the field bioassay, the neonicotinoid clothianidin was generally not as effective as fenthion or alpha-cypermethrin, it is moderately efficacious in controlling adult *B. tryoni*.

When stone fruit was dipped in insecticide and exposed to adult flies, to mimic a cover spray followed by wild flies subsequently moving into the area, the traditional insecticides used in *B. tryoni* control, the organophosphates dimethoate (half-label rate) and fenthion (full-label rate), showed the highest efficacy. Nonetheless, when fruit and adult flies received direct topical application, alpha-cypermethrin had the quickest mortality rate, followed closely by clothianidin. However, the production of offspring, whether fruit was sprayed first with clothianidin and exposed to adult flies, or infested fruit was treated with insecticide, may be a concern given the nil tolerance for *B. tryoni* detections by most importing markets. Clothianidin demonstrated a moderate field efficacy; however, the efficacy of alpha-cypermethrin was close to that of fenthion. Although trichlorfon performed well initially when fruit was dipped in insecticide and then exposed to adult flies, its residual activity against adults was poor after only one day, although few offspring were produced across all residuals. Similarly, abamectin, which showed initial potential in bioassay 1, performed quite poorly during longer residual periods against adults, although low numbers of offspring were produced. The efficacy of abamectin and trichlorfon warrant further testing. In the laboratory, emamectin benzoate caused a 100% kill rate when applied topically, an 88% efficacy when adults were exposed to fruit dipped in insecticide, a residual activity comparable to that of fenthion (full-rate), a reasonable speed of kill, and low numbers of offspring. However, when trialled in the field, its efficacy was very poor for both 1 and 5 day residuals (<50%), suggesting that females were able to successfully oviposit in pesticide treated fruit, and that offspring were able to complete development.

Infested fruit treated with fenthion (half- and full-label rate) and the chloronicotinyl insecticides acetamiprid and thiacloprid, showed a very good efficacy against developing offspring. Yee and Alston [28] sprayed Western cherry fruit fly, *Rhagoletis indifferens*, infested cherries with spinosad, spinosad bait, imidacloprid, and thiacloprid. The latter two insecticides produced significantly fewer live larvae than other treatments after eight days and all reduced larval emergence over 30 days. The authors suggested that imidacloprid in particular may be useful in replacing dimethoate as a post-harvest spray. Similarly, the present study suggests that thiacloprid and acetamiprid may be useful as a post-harvest spray for *B. tryoni*.

Historically, malathion has been the most frequently used insecticide against fruit flies [29]. In Australia, it is often used as the toxicant in bait sprays. Although malathion use is currently restricted in many countries due to concerns over its poor selectivity and negative impacts on human health [30], it is the predominant alternative to dimethoate and fenthion for fruit fly control listed by the Australian government [22]. Although it performed reasonably well in bioassay 1, there were several chemicals that performed better, and due to this and increasing concerns over its long term viability, malathion was not tested further.

Despite encouraging results in the laboratory bioassays, emamectin benzoate showed poor efficacy against both adults and the resulting development of offspring in the field trial. Emamectin benzoate, like all avermectins, does not exhibit a rapid knockdown activity against insects; however, paralysis is rapid upon ingestion [31]. Although avermectins intoxicate arthropods via both contact and ingestion, the latter is thought to be the main route whereby arthropods accumulate a lethal dose. Emamectin benzoate was developed for the control of lepidopterous pests [31] and there are no published studies of its use for the Tephritidae. It has been tested against other Dipterans including *Liriomyza trifolii* Burgess [32] and *Musca domestica* Linnaeus [33], with some success. However, emamectin benzoate appears to be generally less toxic to most non-lepidopterous arthropods compared with lepidopterous arthropods [31].

The pyrethroid alpha-cypermethrin was not tested in the laboratory beyond bioassay 1, due to its slightly poorer performance compared with several other insecticides when fruit was dipped and then exposed to *B. tryoni*. However, its efficacy when topically applied (100%) led to the inclusion of this chemical in the field trial, where it demonstrated a very good efficacy, which was not dissimilar to that of fenthion (full-rate). A laboratory study that compared the mortality of *R. indifferens* exposed to malathion, spinosad, and zeta-cypermethrin, demonstrated that zeta-cypermethrin was more effective, causing up to 100% mortality after 2 h of exposure [34]. Further, when flies walked on dried zeta-cypermethrin residues for 20–25 min, or were directly sprayed, and then exposed to cherries with dried residues, simulating the exposure of mature female flies in a treated orchard, zeta-cypermethrin prevented all oviposition. This was not the case for malathion and spinetoram [34]. The efficacy of the topical application of alpha-cypermethrin at a range of doses was compared between twenty populations of the olive fruit fly, *Bactrocera oleae* (Rossi), from mainland Greece and its islands [35]. It was concluded that cypermethrin is effective against *B. oleae*, with variations observed among populations in their response to the pyrethroid, with ED50 values ranging from 0.14 to 3.28 ng insect−1.

The neo-nicotinoid clothianidin had a delayed adult fly mortality effect in the laboratory bioassays. In a laboratory study, Chuang and Hou [36] showed that the mortality of the male oriental fruit fly, *Bactrocera dorsalis* (Hendel), using the traditional toxicant, naled, was 98.3–100% at 24–72 h after treatment, whereas the neonicotinoid insecticides imidacloprid and acetamiprid caused only 60–80% at 24–72 h after treatment. Similarly, clothianidin had a delayed lethal effect of 80% and 91.8% male mortality after 24 h and 72 h, respectively [36]. Rapid kill times are desirable, as flies may be able to oviposit after exposure to an insecticide if mortality is delayed. Further, in the field, clothianidin was more effective when used without a surfactant than when applied with a surfactant, regardless of the residual period, although it is not clear why this might be the case.

The results of this work enabled the justification and application by AKC Consulting for a permit for the use of clothianidin in nectarines, peaches, apricots, and plums against *B. tryoni*, submitted by Growcom on behalf of Summerfruit Australia Ltd with the APVMA in May 2012. The use pattern on the requested permit is identical to the existing label with respect to the number of applications and rates. However, a shorter 7-day withholding period was requested, rather than the current 21 day withholding period on the label. In September 2013, the APVMA issued a permit (PER14252) [37] with the shorter withholding period for the use of Clothianidin in persimmon, pome, and stone fruit against *Bactrocera* and *Ceratitis* species. Further study should determine how clothianidin may be incorporated in a spray regime as part of an integrated pest management program under different climatic scenarios and across a range of fruit types, both with and without a surfactant. Similarly, the results of the current project have informed the justification and application submitted by Growcom on behalf of Summerfruit Australia Ltd. for a minor use permit (PER14875) for the use of registered products containing 100-g/L alpha-cypermethrin on stone fruit (except cherries) against *B. tryoni* and the Meditteranean fruit fly, *Ceratitis capitata* (Wiedemann) [38], a pest in Western Australia. Further, anecdotal field evidence suggests that alpha-cypermethrin is very effective, while clothianidin has variable results [39].

Van der Sluijs et al. [40] have conducted an extensive review on the effects of the neonicotinoids, including clothianidin, on bees and pollinator services, and have concluded that a move to pollinator friendly alternatives to neonicotinoids is urgently needed for the sake of the sustainability of pollinator ecosystem services. Indeed, the recent decision by the European Commission to temporarily ban the use of clothianidin, imidacloprid, and thiamethoxam in crops attractive to bees follows this course [41]. In Australia, the permit for Sumitomo Samurai Systemic Insecticide (containing: 500 g/L clothianidin as its only active constituent) includes a clause for the protection of bees [37].

Pesticides should be considered one “tool” in an integrated pest management approach to managing *B. tryoni*. Worldwide, many successful tephritid management programs rely upon a combination of in-field techniques, often as part of an area-wide management program [42,43,44,45,46,47]. These include combinations of parasitoids [48,49,50], sterile insect technique (SIT) [42,51,52,53], male annihilation technique [54,55], protein bait sprays [56,57], female-biased attractants [58,59], crop or host sanitation [47,56], and cover sprays [8,60]. In Australia, although chemical cover sprays have played a major role in *B. tryoni* management over the past decades, we are seeing programs that increasingly rely on SIT, MAT, and protein bait spray [2].

## 5. Conclusions

Alpha-cypermethrin may be a suitable control option for the management of *B. tryoni*. Although not registered against fruit fly, alpha-cypermethrin showed an efficacy not unlike that of fenthion in the field, and is registered in stone fruit and a range of other crops against other pests, and is therefore a feasible candidate for fruit fly control. Clothianidin may be useful as part of a spray regime that is part of an integrated pest management program; however, further testing of this chemical under a range of different climatic conditions and across different fruits is required, both with and without a surfactant. Clothianidin plus MAX surfactant costs approximately AU$79.90/ha, while alpha-cypermethrin is approximately AU$6.61/ha; the latter may therefore be more economically viable. Fenthion at a half-label rate showed potential, and with further rate testing, it may have been possible to reduce the rate to a level that will provide effective *B. tryoni* control; however, this product can no longer be used or supplied in Australia. Further laboratory and field testing are warranted for abamectin and trichlorfon. Acetamiprid and thiacloprid showed a very good efficacy against developing offspring, and could be considered as post-harvest treatments for *B. tryoni* after further testing.

## Figures and Tables

**Table 1:** The selected trial chemicals, trade name, supplier/manufacturer, recommended rate, trial rate, and wetting agents for *Bactrocera tryoni* pesticide application.

Pesticide (Active Constituent)	Trade Name	Supplier/Manufacturer	Recommended Spray Volume (Label Rate or As Recommended by Supplier)	Trial Rate	Wetting Agent (As Per Label)
Abamectin (18 g/L)	Vertimec^®^	Syngenta^®^, Macquarie Park, Australia	600 mL/ha	0.6 mL/L	
Acetamiprid (20%)	Acetamiprid 20SL	Makhteshim Chemical Works Ltd., Beer Sheva, Israel	550–810 gai/ha	0.81 g/L	
Alpha-cypermethrin (100 g/L)	Astound^®^	Nufarm Australia Ltd., Laverton North, Australia	100 mL/100L	1.0 mL/L	
Bifenthrin (250 g/L)	Talstar 250 EC	FMC Australasia Pty Ltd., Murarrie, Australia	32 mL/100L	0.32 mL/L	
Clothianidin (500 g/Kg)	Sumitomo Samurai Systemic Insecticide^TM^	Sumitomo Chemical Australia Pty Ltd., Epping, Australia	40 g/100L	0.4 g/L	The addition of MAXX Organosilicone Surfactant at 50 mL/100 L water may improve efficacy.
Cyantraniliprole (100 g/L)	Exirel^TM^	DuPont^TM^, Wilmington, NC, USA	0.3 gai/L	0.3 gai/L	
DC-092 Coded Molecule (not specified)	n/a	Bayer Crop Science, East Hawthorne, Australia	120 mL/100L	1.2 mL/L	
Dimethoate—half rate (100 g/L)	Danadim^TM^	Ospray, Cottonvale, Australia	75 mL/100L (Full rate)	0.375 mL/L (Half rate)	
Emamectin (44 g/kg) present as Emamectin benzoate	Proclaim^®^	Syngenta^®^, Macquarie Park, Australia	250–300 g/ha	0.3 g/L	
Fenthion—full rate (550 g/L)	Lebaycid^®^	Bayer Crop Science, Hawthorne, Australia	75 mL/100L	0.75 mL/L	
Fenthion—half rate (550 g/L)	Lebaycid^®^	Bayer Crop Science, Hawthorne, Australia	75 mL/100L	0.375 mL/L	
Imidacloprid (200 g/L)	Confidor^®^ 200 SC	Bayer Crop Science, Hawthorne, Australia	120 mL/100L	1.2 mL/L	
Malathion (30% w/w) (Experimental product)	Malathion CS	Cheminova A/S, Lemvig, Denmark	97.5 mL/100L	0.975 mL/L	
Methomyl (225 g/L)	Farmoz Electra^®^ 225	FarmOz, St Leonards, Australia	200 mL/ha	0.2 mL/L	
Spinetoram (250 g/Kg)	Delegate™	Dow AgroSciences, Indianapolis, IN, USA	20 gpr/Hl	0.2 g/L	Addition of a non-ionic wetting agent at its label rate, such as Agral^®^ at 10 mL/100 L, may improve control under less than ideal application conditions.
Tau-fluvalinate (240 g/L)	Farmoz Klartan Insecticide^®^	FarmOz, St Leonards, Australia	20 mL/100L	0.2 mL/L	
Thiacloprid (480 g/L)	Calypso^®^ 480 SC	Bayer Crop Science, Hawthorne, Australia	50 mL/100L	0.5 mL/L	Add a non-ionic wetting agent at 10 mL/100 L regardless of whether applying by dilute or concentrate spraying.
Trichlorfon (500 g/L)	Lepidex 500	Nufarm, Laverton North, Australia	250 mL/100L	2.5 mL/L	

**Table 2:** The mean percentage mortality at 24 h, time to reach 50% mortality, and the instantaneous mortality rate (*λ*) of adult *Bactrocera tryoni* exposed to fruit dipped in insecticide (bioassay 1). Weibull’s shape parameter ***α*** = 0.55.

Insecticide	Mean (%) Mortality at 24 h	Time to 50% Mortality (95% Fiducial Limits) (min)	*Λ* (Mortality Rate Per min)
Abamectin	86.7 ab	275 (168–450)	0.0313 de
Acetamiprid	43.3 cde	78 (28–215)	0.0627 bc
Alpha-cypermethrin	73.3 abc	190 (85–430)	0.0382 cd
Bifenthrin	15.0 efg	14,000	0.0036 gh
Clothianidin	80.0 ab	147 (72–300)	0.0442 cd
Cyantraniliprole	8.3 fg	60,000	0.0016 h
DC-092 Coded molecule	0.0 g	n/a	n/a
Dimethoate (Half-label rate)	100.0 a	17 (9–34)	0.1438 a
Emamectin benzoate	88.3 ab	210 (105–420)	0.0363 d
Fenthion (Half-label rate)	98.3 a	98 (50–192)	0.0552 bc
Fenthion (Standard; full-rate)	98.3 a	50 (27–100)	0.0784 b
Imidacloprid	10.0 fg	40,000	0.002 h
Maldison/malathion	60.0 bcd	590 (275–1300)	0.0206 e
Methomyl	36.7 def	2200	0.0097 f
Spinetoram	58.3 bcd	3050	0.0083 fg
Tau-fluvalinate	13.3 efg	70,000	0.0015 h
Thiacloprid	20.0 efg	9500	0.0044 gh
Trichlorfon	90.3 a	70 (35–140)	0.0663 b

Within each column, values followed by the same letter are not significantly different from one another (*p* > 0.05). Means are back transformed from logit means.

**Table 3:** The mean percentage mortality at 24 h, time to reach 50% mortality, and the instantaneous mortality rate (*λ*) of adult *Bactrocera tryoni* exposed to a topical application of insecticide (bioassay 1). Weibull’s shape parameter ***α*** = 0.79.

Insecticide	Mean (%) Mortality at 24 h	Time to 50% Mortality (95% Fiducial Limits) (min)	*Λ* (Mortality Rate Per minute)
Abamectin	100.0 a	53 (36–80)	0.0300 h
Acetamiprid	100.0 a	14 (8–23)	0.0864 efg
Alpha-cypermethrin	100.0 a	5 (2.7–7.5)	0.2083 a
Bifenthrin	100.0 a	12 (7–20)	0.0974 def
Clothianidin	100.0 a	6 (3.5–10)	0.166 ab
Cyantraniliprole	18.3 b	3900 (1600–9350)	0.001 j
DC-092 Coded molecule	38.3 b	1850 (960–3500)	0.0018 j
Dimethoate(reduced rate)	100.0 a	19 (11–32)	0.0661 g
Emamectin benzoate	100.0 a	97 (53–145)	0.0202 h
Fenthion(Reduced rate)	100.0 a	16 (9–25)	0.0793 fg
Fenthion(Standard)	100.0 a	8 (5–13)	0.1331 bcd
Imidacloprid	100.0 a	400 (230–690)	0.0061 i
Maldison/malathion	100.0 a	8 (4.5–13)	0.1397 bc
Methomyl	100.0 a	7 (4-11.5)	0.1511 abc
Spinetoram	100.0 a	86 (52–142)	0.0206 h
Tau-fluvalinate	98.3 a	9 (5–15)	0.1267 bcde
Thiacloprid	96.7 a	77 (46–130)	0.0224 h
Trichlorfon	100.0 a	10 (6–17)	0.1109 cdef

Within each column, values followed by the same letter are not significantly different from one another (*p* > 0.05). Means are back transformed from logit means.

**Table 4:** The mean percentage mortality at 24 h, time to reach 50% mortality, and the instantaneous mortality rate (*λ*) of adult *Bactrocera tryoni* exposed to filter paper dipped in insecticide (bioassay 1). Weibull’s shape parameter ***α*** = 0.52.

Insecticide	Mean (%) Mortality at 24 h	Time to 50% Mortality (95% Fiducial Limits) (min)	*Λ* (Mortality Rate Per minute)
Abamectin	38.3 de	3700 (1650–8000)	0.0098 h
Acetamiprid	16.7 ef	1600 (845–7500)	0.0149 gh
Alpha-cypermethrin	90.0 ab	47 (18–120)	0.0939 bc
Bifenthrin	80.0 abc	145 (57–400)	0.0515 de
Clothianidin	81.7 abc	64 (33–210)	0.0695 cd
Cyantraniliprole	1.7 f	1,800,000	0.0004 j
DC-092 Coded molecule	1.7 f	1,800,000	0.0004 j
Dimethoate (Half-label rate)	96.7 a	47 (18–120)	0.0934 bc
Emamectin benzoate	56.7 cd	1310 (500–3600)	0.0166 g
Fenthion (Half-label rate)	100.0 a	24 (10–60)	0.1333 ab
Fenthion (Standard; full-rate)	100.0 a	105 (40–250)	0.0619 d
Imidacloprid	16.7 ef	2400 (500–12500)	0.012 gh
Maldison/malathion	66.7 bcd	440 (160–1150)	0.0294 ef
Methomyl	61.2 cd	495 (182–1350)	0.0275 f
Spinetoram	15.0 ef	25,200	0.0036 i
Tau-fluvalinate	11.7 ef	2400 (800–7000)	0.0122 gh
Thiacloprid	11.7 ef	20,500	0.004 i
Trichlorfon	100.0 a	15 (6–36)	0.1726 a

Within each column, values followed by the same letter are not significantly different from one another (*p* > 0.05). Means are back transformed from logit means.

**Table 5:** The mortality of adult *Bactrocera tryoni*, exposed to stone fruit dipped in insecticide and aged for 0, 1, 3, or 5 days and then allowed to oviposit, and the resulting offspring (bioassay 2).

Treatments	Adult mortality			Offspring (Mean No. Per Whole Fruit)		
	1440 min	2880 min	4320 min	Larvae (dead)	Pupae	Adults (F1)
Water (control)	1.5 j	1.9 n	2.3 i	0.00 f	115.6 a	110.3 a
Abamectin 0	51.7 ef	80.8 abcdef	95.8 b	14.9 a	1.6 fg	0.0 h
Abamectin 1	22.5 gh	61. 7 fgh	80.0 bcd	3.4 c	2.8 fg	0.6 g
Abamectin 3	1.7 j	35.0 ij	58.3 de	1.7 d	2.7 fg	0.3 g
Abamectin 5	0.8 j	20.0 jkm	65.0 cde	4.0 c	0.6 ghi	0.0 h
Acetamiprid 0	1. 7 j	10.8 km	16.7 gh	0.1 e	20.2 c	18.8 c
Acetamiprid 1	4.2 ij	12.5 km	16.7 gh	0.0 f	46.1 b	45.1 b
Acetamiprid 3	2.5 j	17.5 jkm	26.7 fg	0.0 f	22.0 c	20.7 c
Acetamiprid 5	1. 7 j	4.2 mn	5.8 hi	0.3e	13.1 d	12.9 d
Clothianidin 0	67.5 bcde	82.5 abcdef	90.8 bc	0.4 e	8.6 e	8.4 e
Clothianidin 1	62.5 cde	85.8 abcde	93.3 bc	0.0 f	2.4 fg	2.3 f
Clothianidin 3	29.2 fgh	46. 7 ghi	65.0 cde	0.3 e	8.0 e	7.6 e
Clothianidin 5	48.3 ef	65.0 efg	84.2 bc	0.1 e	0.4 hi	0.3 g
Emamectin benzoate 0	91. 7 a	96. 7 a	100.0 a	7.2 b	1.3 ghi	0.3 g
Emamectin benzoate 1	80.0 abc	93.3 ab	96.7 b	13.9 a	0.7 ghi	0.1 g
Emamectin benzoate 3	66. 7 bcde	85.8 abcde	95.8 b	7.0 b	0.3 g	0.1 g
Emamectin benzoate 5	45.8 efg	71.7 def	87.5 bc	1.4 d	0.3 hi	0.1 g
Fenthion (Full-label rate) 0	82.5 abc	92.5 abc	97.5 b	0.0 f	0.0 j	0.0 h
Fenthion (Full-label rate) 1	85.0 ab	94.2 ab	96.7 b	0.0 f	0.0 j	0.0 h
Fenthion (Full-label rate) 3	76.7 abcd	93.3 ab	96.7 b	0.0 f	0.0 j	0.0 h
Fenthion (Full-label rate) 5	52.5 ef	74.2 cdef	90.0 bc	0.0 f	0.0 j	0.0 h
Fenthion (Half-label rate) 0	80.8 abc	95.0 a	99.2 ab	0.0 f	0.3 i	0.2 g
Fenthion (Half-label rate) 1	60.8 cde	76.7 bcdef	92.5 bc	0.0 f	0.0 j	0.0 h
Fenthion (Half-label rate) 3	44.2 efg	66.7 defg	87.5 bc	0.0 f	0.5 ghi	0.5 g
Fenthion (Half-label rate) 5	55.8 de	70.8 defg	86.7 bc	0.0 f	0.0 j	0.0 h
Trichlorfon 0	80.8 abc	87.5 abcd	90.0 bc	0.0 f	0.8 ghi	0.7 g
Trichlorfon 1	29.2 fgh	36.7 hij	52.5 ef	0.0 f	0.4 hi	0.3 g
Trichlorfon 3	15.0 hij	18.3 jkm	23.3 gh	0.1 e	2.2 f	2.2 f
Trichlorfon 5	19.2 hi	30.8 ijk	39.2 efg	0.0 f	0.3 i	0.3 g

Within each column, values followed by the same letter are not significantly different from one another (*p* > 0.05). Means are back transformed from logit means.

**Table 6:** Mean *Bactrocera tryoni* offspring produced from fruit exposed to adults for 48 h, and then sprayed with insecticide (bioassay 3).

Treatments	Larvae (Dead)	Pupae	Adults (F1)
Water (control)	0.22 b	30.96 a	29.48 a
Acetamiprid	0.00 c	0.04 d	0.04 d
Clothianidin	0.26 b	0.59 c	0.56 c
Emamectin benzoate	3.15 a	4.26 b	1.74 b
Fenthion(Standard; Full-label Rate)	0.00 c	0.00 d	0.00 d
Fenthion (Half-label Rate)	0.04 c	0.00 d	0.00 d
Imidicloprid	0.00 c	0.44 c	0.26 c
Thiacloprid	0.93 b	0.00 d	0.00 d

Note: Within a column, values followed by the same letter are not significantly different from one another (*p* > 0.05). Means are back transformed from logit means.

**Table 7:** Mortality of adult *Bactrocera tryoni*, after 48 h exposed to one- and five-day old field aged residues of insecticides applied to peach trees, and subsequent offspring.

Treatment	Days Sprayed	Adult Mortality (%)	Offspring (Mean No. Per Whole Fruit)		
			Larvae (Dead)	Pupae	Adults (F1)
Water (control)	1	0.6 g	47.4 a	69.8 ab	61.4 ab
	5	0.6 g	13.8 abc	164.3 a	154.7 a
Alpha-cypermethrin	1	97.2 ab	0.0 d	0.0 f	0.0 f
	5	98.8 ab	0.0 d	0.0 f	0.0 f
Clothianidin	1	70.9 cd	3.6 abc	3.7 cd	3.2 cd
	5	84.0 bc	0.3 bc	0.9 cde	0.9 cde
Clothianidin + Surfactant	1	71.5 cd	0.0 d	0.0 f	0.0 f
	5	57.7 de	0.5 abc	6.5 cd	6.5 cd
Emamectin benzoate	1	37.7 e	18.4 ab	0.0 f	0.0 f
	5	17.8 f	37.6 a	14.1 bc	10.7 bc
Fenthion (half-label rate)	1	99.4a	0.0 d	0.3 de	0.3 de
	5	86.4 bc	0.0 d	0.0 f	0.0 f
Fenthion (full-label rate)	1	100.0 a	0.2 c	0.2 e	0.2 e
	5	100.0 a	0.0 d	0.0 f	0.0 f

Within a column and within days sprayed, values followed by the same letter are not significantly different from one another (*p* > 0.05). Means are back transformed from logit means.
